# Abnormal myocardial perfusion reserve and myocardial infarction determine cardiovascular outcomes in type 2 diabetes mellitus

**DOI:** 10.1093/ehjci/jeag047

**Published:** 2026-02-25

**Authors:** Noor Sharrack, Kristopher D Knott, Jian L Yeo, Tushar Kotecha, Louise A E Brown, Aldostefano Porcari, Robert D Adam, Gaurav S Gulsin, Sharmaine Thirunavukarasu, Amrit Chowdhary, Masafumi Takafuji, Eylem Levelt, John P Greenwood, David L Buckley, Theresa Munyombwe, Chris P Gale, Peter Kellman, James C Moon, Marianna Fontana, Gerry P McCann, Peter P Swoboda, Sven Plein

**Affiliations:** Leeds Institute of Cardiovascular and Metabolic Medicine, University of Leeds, Leeds LS2 9NL, United Kingdom; Barts Heart Centre, St Bartholomew’s Hospital, London, United Kingdom; Institute of Cardiovascular Science, University College London, London, United Kingdom; Department of Cardiovascular Sciences, University of Leicester and the NIHR Leicester Biomedical Research Centre, Glenfield Hospital, Leicester, United Kingdom; Institute of Cardiovascular Science, University College London, London, United Kingdom; Division of Medicine, National Amyloidosis Centre, University College London, Royal Free Campus, London, United Kingdom; Leeds Institute of Cardiovascular and Metabolic Medicine, University of Leeds, Leeds LS2 9NL, United Kingdom; Division of Medicine, National Amyloidosis Centre, University College London, Royal Free Campus, London, United Kingdom; Cardiovascular Department, Centre for Diagnosis and Treatment of Cardiomyopathies, Azienda Sanitaria Universitaria Giuliano-Isontina (ASUGI), University of Trieste, Trieste, Italy; Barts Heart Centre, St Bartholomew’s Hospital, London, United Kingdom; Institute of Cardiovascular Science, University College London, London, United Kingdom; Institute of Cardiovascular Diseases, Carol Davila University of Medicine and Pharmacy, Bucharest, Romania; Department of Cardiovascular Sciences, University of Leicester and the NIHR Leicester Biomedical Research Centre, Glenfield Hospital, Leicester, United Kingdom; Leeds Institute of Cardiovascular and Metabolic Medicine, University of Leeds, Leeds LS2 9NL, United Kingdom; Leeds Institute of Cardiovascular and Metabolic Medicine, University of Leeds, Leeds LS2 9NL, United Kingdom; Leeds Institute of Cardiovascular and Metabolic Medicine, University of Leeds, Leeds LS2 9NL, United Kingdom; Department of Radiology and Mie University Hospital, Tsu, Mie, Japan; Leeds Institute of Cardiovascular and Metabolic Medicine, University of Leeds, Leeds LS2 9NL, United Kingdom; Leeds Institute of Cardiovascular and Metabolic Medicine, University of Leeds, Leeds LS2 9NL, United Kingdom; Department of Cardiology, Leeds General Infirmary, Leeds Teaching Hospitals NHS Trust, Great George Street, Leeds LS1 3EX, UK; Leeds Institute of Cardiovascular and Metabolic Medicine, University of Leeds, Leeds LS2 9NL, United Kingdom; Leeds Institute of Cardiovascular and Metabolic Medicine, University of Leeds, Leeds LS2 9NL, United Kingdom; Leeds Institute for Data Analytics, University of Leeds, Leeds, United Kingdom; Leeds Institute of Cardiovascular and Metabolic Medicine, University of Leeds, Leeds LS2 9NL, United Kingdom; Department of Cardiology, Leeds General Infirmary, Leeds Teaching Hospitals NHS Trust, Great George Street, Leeds LS1 3EX, UK; Leeds Institute for Data Analytics, University of Leeds, Leeds, United Kingdom; Department of Health and Human Services, National Heart, Lung, and Blood Institute, National Institutes of Health, Bethesda, MD, USA; Barts Heart Centre, St Bartholomew’s Hospital, London, United Kingdom; Institute of Cardiovascular Science, University College London, London, United Kingdom; Institute of Cardiovascular Science, University College London, London, United Kingdom; Division of Medicine, National Amyloidosis Centre, University College London, Royal Free Campus, London, United Kingdom; Department of Cardiovascular Sciences, University of Leicester and the NIHR Leicester Biomedical Research Centre, Glenfield Hospital, Leicester, United Kingdom; Leeds Institute of Cardiovascular and Metabolic Medicine, University of Leeds, Leeds LS2 9NL, United Kingdom; Leeds Institute of Cardiovascular and Metabolic Medicine, University of Leeds, Leeds LS2 9NL, United Kingdom; Department of Cardiology, Leeds General Infirmary, Leeds Teaching Hospitals NHS Trust, Great George Street, Leeds LS1 3EX, UK

**Keywords:** quantitative myocardial perfusion, T2DM, CMR, cardiovascular outcomes, coronary microvascular dysfunction

## Abstract

**Aims:**

In individuals with type 2 diabetes mellitus (T2DM), both myocardial ischemia and myocardial infarction (MI) are associated with adverse cardiovascular outcomes. The incremental prognosis of both risks is unknown. We aimed to investigate whether abnormal myocardial perfusion reserve (MPR), as a surrogate marker for ischemia and presence of MI offers incremental prognostic value in predicting major adverse cardiovascular and cerebrovascular events (MACCE) in patients with T2DM.

**Methods and results:**

A retrospective multicentre cohort of 572 individuals with T2DM and healthy controls underwent quantitative stress myocardial perfusion cardiovascular magnetic resonance (CMR) to determine MPR and late gadolinium enhancement (LGE) to identify MI. Patients were divided into three groups: MI- and normal MPR, MI+ or abnormal MPR and MI+ and abnormal MPR. Cox proportional hazard models quantified associations between MPR and MI with MACCE (composite of all-cause death, MI, stroke, heart failure hospitalization, and late coronary revascularization>90 days after the CMR scan). Over a median of 28 months (IQR 25–31 months), 81 participants (14%) accrued at least one MACCE, including 25 (4%) deaths. Presence of either abnormal MPR or MI was associated with increased MACCE (MI- and normal MPR: 8% MACCE; MI+ or abnormal MPR: 15% MACCE (adjusted HR compared with normal 1.86 (95% CI 1.06–3.25, *P* = 0.03)); presence of both MI and abnormal MPR had the highest event rate: 30% MACCE (adjusted HR compared with normal 3.24 (95% CI 1.75–6.01, *P* < 0.001)).

**Conclusion:**

In T2DM, abnormal MPR or MI are associated with MACCE, and the presence of both offers incremental prognostic value.

## Graphical Abstract

**Graphical Abstract:**
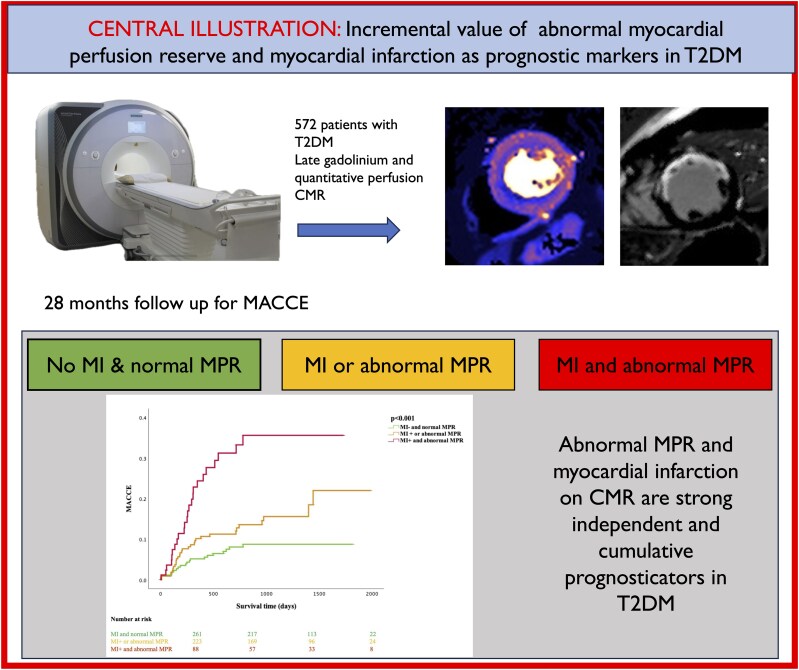
In patients with T2DM, direct quantification of global MPR as a surrogate measure of underlying ischemia, in addition to detection of MI using CMR, is an independent marker of cardiovascular and cerebrovascular outcomes.

**See the editorial comment for this article ‘Quantitative perfusion mapping in diabetic patients’, by J. Garot and S. Duhamel, https://doi.org/10.1093/ehjci/jeag048.**

## Introduction

Individuals with T2DM are at an increased risk of cardiovascular disease, including epicardial coronary artery disease (CAD), myocardial infarction (MI), and coronary microvascular dysfunction (CMD), which are associated with adverse clinical outcomes.^1–4^ International practice guidelines recommend non-invasive imaging for risk stratification of patients with diabetes mellitus (DM) and CAD,^1^ including the detection of ischemia and silent MI, which have shown independent prognostic relevance.^5–8^

Several studies have demonstrated that global myocardial ischemia is associated with adverse outcomes in patients with DM.^8–10^ Most of these previous studies were based on myocardial perfusion Positron Emission Tomography (PET) using myocardial blood flow reserve (MBFR) as a surrogate marker for ischemia and CMD.^11^ Myocardial perfusion can also be accurately quantified using myocardial perfusion stress cardiovascular magnetic resonance (CMR), which is becoming more widely used in clinical routine. This method has been shown to have prognostic value in unselected patients with suspected CAD, including a subset of patients with DM.^10,12^ However, quantitative myocardial perfusion by CMR using myocardial perfusion reserve (MPR) as a surrogate marker of global ischemia or CMD has not yet been validated as a prognostic marker in patients with T2DM.

CMR is also the reference standard for the detection of MI, using late gadolinium enhancement (LGE).^13–15^ In a study of patients with DM, the presence of silent MI on LGE was linked with a more than threefold increase in the risk of major adverse cardiovascular events (MACE).

CMR allows routine concurrent quantification of myocardial perfusion and silent MI within a single non-invasive imaging test, at high spatial resolution, relatively low cost compared to PET and without exposure to ionizing radiation. In this study, we established the individual and combined prognostic value of abnormal MPR and the presence of MI as determined by CMR in a contemporaneous multi-centre population of patients with T2DM compared to healthy controls.

## Methods

### Patients

This was a retrospective analysis of 572 patients aged 18 years or older recruited from four United Kingdom (U.K.) centres (St Bartholomew’s Hospital, Barts Heart Centre, London; Glenfield Hospital, University Hospitals of Leicester NHS Trust, Leicester; Leeds Teaching Hospitals NHS Foundation Trust, Leeds and the Royal Free Hospital, Royal Free London NHS Foundation Trust, London) between September 2016 and May 2021. All patients had T2DM, based on a HbA1c ≥6.5% (≥48 mmol/L) or a known diagnosis of T2DM. Patients with other types of DM were not included. Two-thirds of the patients (68%) were on Metformin treatment, 20% on Gliclazide, 15% on Insulin, 13% on GLP-1, 9% on SGLT2-inhibitors and 3% on Gliptins. Exclusion criteria included contraindications to adenosine, gadolinium-based contrast or Magnetic Resonance Imaging (MRI).

All patients had undergone quantitative myocardial perfusion stress and LGE CMR using comparable imaging protocols for clinical or research indications (Supplementary data online, *Table S1*). Those with sub-optimal image quality, significant mis-triggering or other artefacts were excluded.

### Controls

In order to derive cut-off values for normal myocardial perfusion, we recruited 52 age and sex-matched, healthy controls with no history of cardiac disease or major risk factors for cardiovascular disease from two of the four cardiac centres (Leeds Teaching Hospitals NHS Foundation Trust, Leeds and Glenfield Hospital, University Hospitals of Leicester NHS Trust, Leicester) between September 2016 and May 2021. Exclusion criteria included a past medical history of hypertension, hypercholesterolemia, DM, smoking, previous CAD or revascularization, transient ischaemic attack (TIA) or stroke, regional perfusion defect on index or previous stress CMR, contraindications to adenosine, gadolinium-based contrast or MRI and subsequent evidence of abnormal LGE on CMR.

### Ethical approval and consent to participate

For participants in both the patient and control cohorts, written informed consent was attained for use of their data for research purposes. Approval was provided by the national research ethics service (NRES) for each site and conducted in accordance with the Declaration of Helsinki.

### Clinical outcomes

Patient comorbidities and clinical outcomes were collated from electronic patient records and the National Health Service (NHS) spine portal through deterministic linkage using each patient’s unique NHS identification number. Comorbidities that were recorded included hypertension, dyslipidaemia, atrial fibrillation, CAD, coronary revascularization (percutaneous coronary intervention (PCI) or coronary artery bypass graft (CABG)) surgery, MI, stroke, TIA, peripheral vascular disease and cancer. A minimum of one year of follow-up data was available for all participants.

The primary outcome was a composite of major adverse cardiovascular and cerebrovascular events (MACCE) defined as all-cause death, MI, stroke, heart failure hospitalization, and late coronary revascularization more than 90 days after the CMR scan. Where patients had more than one MACCE, the first event was used.

The definition of MI was type 1 spontaneous MI as defined by the international task force criteria using the third universal definition, ‘myocardial injury related to primary myocardial ischaemia’.^16^ Heart failure was defined as ‘a clinical syndrome consisting of cardinal symptoms (e.g. breathlessness, ankle swelling and fatigue) that may be accompanied by signs (e.g. elevated jugular venous pressure, pulmonary crackles and peripheral oedema), due to structural and/or functional abnormality of the heart’.^17^ Heart failure hospitalization was defined as an admission to hospital with heart failure as the primary diagnosis in the discharge letter through review of patient records.

### Cardiovascular magnetic resonance (CMR) imaging

CMR scans were performed on 1.5T (Magnetom Aera, Siemens Healthcare, Erlangen, Germany) or 3T (Prisma or Skyra, Siemens Healthcare, Erlangen, Germany) scanners with a standard protocol consisting of localizers, short-axis and long-axis cine imaging, quantitative perfusion imaging and LGE imaging as previously described.^18^ Perfusion imaging on 1.5T scanners used a balanced steady state free precession (bSSFP) pulse sequence, and on 3T scanners a fast low angle shot (FLASH) or bSSFP pulse sequence with imaging parameters as previously described.^19^ Of the patients analysed, 30% were scanned on 1.5T and 70% were scanned on 3T. All healthy controls were imaged on 3T scanners. All participants were instructed to abstain from caffeine for 24 h before the study. For perfusion imaging, adenosine was infused for a minimum of 3 min, at a rate of 140 micrograms/kg/min, which was increased up to a maximum of 210 micrograms/kg/min if there was insufficient haemodynamic response (heart rate increase less than 10 bpm) or there was no symptomatic response, in line with standard clinical practice guidelines.^18^ Images were acquired during free breathing over at least 60 dynamics. A minimum duration of 10 min was kept between stress and subsequent rest perfusion acquisitions. Blood pressure and heart rate were collected at regular intervals during adenosine infusions. For each perfusion acquisition, an intravenous bolus (at a dose of 0.05–0.075 mmol/kg) of gadobutrol (Gadovist, Bayer, Leverkusen, Germany) or gadoterate meglumine (Dotarem, Guerbet, Paris, France) was administered at 4–5 mL/s. Perfusion mapping was performed in 3 short-axis sections, using a dual sequence technique combining a low-resolution arterial input function acquisition and a higher resolution myocardial perfusion acquisition as previously described by Kellman *et al*.^19^

### Image analysis

Image analysis was undertaken by experienced operators and overseen by level 3 CMR-accredited members at the primary site. Those with poor image quality were excluded. Measurement of cardiac volume parameters and the presence of LGE were made using cvi42 software (version 5.9.4, Circle Cardiovascular Imaging, Calgary, Canada). Left ventricular systolic and diastolic volume, ejection fraction, and the presence and distribution (ischaemic or non-ischaemic) of LGE were recorded. Non-ischaemic scar was defined as LGE in a mid-myocardial or subepicardial pattern. MI was defined as LGE in a subendocardial or transmural pattern and a coronary distribution. Rest and stress perfusion maps were generated in-line by an automatic artificial intelligence-supported process whereby myocardial blood flow (MBF) is quantified for each pixel of the myocardium in mL/g/min. By averaging all pixel values in the 3 slices, global MBF and global myocardial perfusion reserve (MPR), defined as the ratio of stress to rest MBF, were derived.^19^ Global MBF and MPR included any myocardial tissue with focal enhancement on LGE images, in order to be consistent with previous literature and to simplify adaptation in clinical practice.^12^ Automatically derived perfusion maps were reviewed by experienced observers to exclude data affected by gating and motion correction artefacts and partial volume effects.

### Statistical analysis

**Figure 1:**
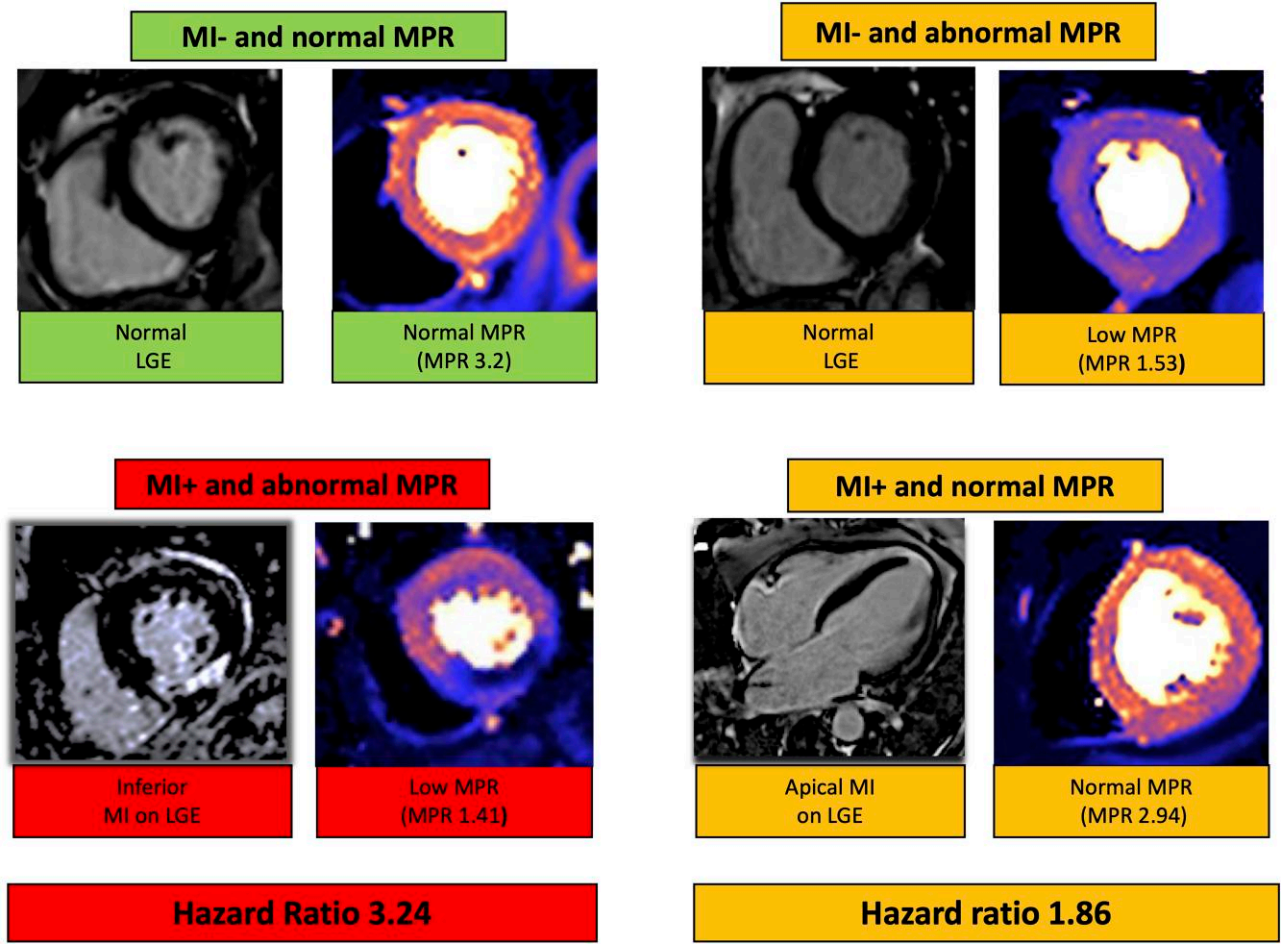
Figure demonstrating example CMR images for MI− and normal MPR, MI+ or abnormal MPR or MI+ and abnormal MPR. LGE, late gadolinium enhancement; MI, myocardial infarction; MPR, myocardial perfusion reserve.

Normality was assessed through the Shapiro-Wilk test, and variance was assessed by Levene’s test for equality of variance. Continuous variables are presented as mean ± SD for parametric data and median and interquartile range for non-parametric data. Categorical variables are presented as absolute values and percentages. Means were compared using the student *t* test for parametric continuous variables, and Mann Whitney U test for non-parametric data. Chi-squared (χ^2^) test was used for categorical variables. The ANOVA test was used for parametric data comparing more than 2 groups, and the Kruskal-Wallis test was used for non-parametric data comparing more than 2 groups. A *P* value of <0.05 was considered statistically significant. As MPR is more reproducible between acquisition and post-processing strategies than MBF, we used the former for the primary analysis of myocardial perfusion. Participant data were categorized depending on threshold MPR values derived using 2 standard deviations below the mean MPR value of the age-matched healthy control group. This threshold was then used to plot Kaplan-Meier hazard curves. We represented data depending on abnormal MPR as defined above and the presence or absence of MI on LGE. Patients with a clinical history of MI, but no matching CMR evidence, were classified as having no MI. Depending on the CMR findings, patients were divided into three distinct groups: MI+ and abnormal MPR, MI+ or abnormal MPR and MI− and normal MPR. *Figure 1* shows example CMR images from each group. Groups were compared using ANOVA with Bonferroni correction. Missing data were managed through listwise deletion of missing data. Univariate Cox regression quantified associations between MPR and MI/ischaemic scar with time to first MACCE. Multivariable Cox regression analysis quantified associations between MPR and MI with time to first MACCE, adjusting for age, sex, left ventricular ejection fraction (LVEF), left ventricular end-diastolic volume (LVEDV), and left ventricular (LV) mass. Net reclassification improvement^20^ and integrated discrimination improvement^21^ methods were used to assess the incremental prognostic value of MPR on LGE MACCE risk prediction. Statistical analysis was performed in SPSS (IBM SPSS Statistics, version 29.0). Additional analysis for NRI was used using JMP (SAS), version 14.2.0.

## Results

A total of 572 patients with T2DM were included with a median follow-up of 28 months (IQR 25–31 months).

### Demographics

#### Patients with T2DM

Patients with T2DM had a mean age of 65 ± 10 years, and 66% were male (*Table 1*). A quarter of patients had reported CAD with previous MI, PCI or CABG surgery (25%). Their mean HbA1c was 59 ± 16 mmol/mol. Angiotensin converting enzyme receptor inhibitors (ACEI), angiotensin receptor blockers (ARB) or angiotensin-receptor neprolysin inhibitors (ARNI) were prescribed in 70% of all patients. Statins were prescribed in 80% of all patients, 68% were prescribed metformin, and 60% were prescribed an antiplatelet agent.

**Table 1:** Patient characteristics divided into groups

Demographics	MI− and normal MPR (n = 261)	MI+ or abnormal MPR (n = 223)	MI+ and abnormal MPR (n = 88)	*P* value	All patients (n = 572)	Healthy Volunteers (n = 52)
**Age (years)**	63 ± 9	64 ± 11	68 ± 10	<0.001*	65 ± 10	64 ± 6
**Male sex**	178 (68)	133 (60)	66 (75)	0.021*	377 (66)	34 (65)
**White**	156 (60)	138 (62)	50 (57)	0.704	344 (60)	N/A
**Asian**	81 (31)	63 (23)	21 (24)	0.425	165 (29)	N/A
**Black**	13 (5)	12 (5)	5 (6)	0.961	30 (5)	N/A
**Other**	11 (4)	9 (4)	12 (14)	0.002*	32 (6)	N/A
**BSA (m^2^)**	2.00 ± 0.2	1.97 ± 0.2	1.90 ± 0.3	0.003*	2.0 ± 0.3	N/A
**Previous PCI/CABG/MI**	18 (7)	76 (34)	48 (55)	<0.001*	142 (25)	0
**Current Smoker**	45 (17)	48 (22)	36 (41)	<0.001*	129 (23)	0
**HTN**	109 (42)	152 (68)	67 (76)	<0.001*	328 (58)	0
**Hypercholesterolemia**	155 (60)	135 (61)	49 (56)	0.716	339 (60)	0
**Previous stroke/TIA**	12 (5)	11 (5)	11 (13)	0.018*	34 (6)	0
**AF**	21 (8)	41 (18)	16 (18)	0.002*	78 (14)	0
**PVD**	9 (4)	18 (8)	17 (19)	<0.001*	44 (8)	0
**HbA1C % (mmol/mol)**	7.5 ± 3.7 (58 ± 17)	7.5 ± 3.5 (59 ± 15)	7.6 ± 3.5 (60 ± 15)	0.662	7.5 ± 3.6 (59 ± 16)	N/A
**Cholesterol (mmol/L)**	4.2 ± 1.5	4.81 ± 1.6	4.17 ± 2	0.190	4.58 ± 1.4	N/A

*P* value is considered significant at <0.05 and indicated by*. ANOVA is used for parametric data, and Kruskal-Wallis for non-parametric data. Continuous variables are presented as mean ± SD or median + IQR, depending on normality. Dichotomous variables are presented as a number (%).

MI, myocardial infarction; MPR, myocardial perfusion reserve; BSA, body surface area; PCI, percutaneous coronary intervention; CABG, coronary artery bypass graft; HTN, hypertension; TIA, transient ischaemic attack; AF, atrial fibrillation; PVD, peripheral vascular disease; LGE, late gadolinium enhancement; N/A, not available.

#### MI− and normal MPR T2DM patients

Patients with T2DM who had no MI and normal MPR on CMR were younger than patients who were MI+ and/or had abnormal MPR. They had a lower prevalence of CAD, hypertension, previous stroke, previous TIA, atrial fibrillation, and peripheral vascular disease compared to the other groups. They had the lowest rate of previous PCI, CABG surgery or MI amongst all the groups. Compared to the other groups, they were less frequently prescribed antiplatelet agents, heart failure medications (including beta blockers, ACEI, ARB, ARNI, mineralocorticoid receptor antagonists (MRA) or diuretics) or insulin (*Table 2*).

**Table 2:** Medications taken by patients divided into groups

Medication	MI− and normal MPR (n = 261)	MI+ or abnormal MPR (n = 223)	MI+ and abnormal MPR (n = 88)	*P* value	All patients (n = 572)
**Aspirin/other antiplatelet**	68 (43)	115 (57)	69 (78)	<0.001*	343 (60)
**DOAC/Warfarin**	15 (10)	34 (17)	13 (15)	0.134	75 (13)
**Statin**	196 (75)	182 (82)	76 (86)	0.053	454 (80)
**Beta blocker**	85 (33)	160 (72)	78 (89)	<0.001*	323 (57)
**ACEI/ARB/ARNI**	145 (56)	173 (78)	78 (89)	<0.001*	398 (70)
**MRA**	10 (4)	35 (16)	19 (22)	<0.001*	64 (11)
**Diuretics**	48 (18)	72 (33)	35 (40)	<0.001*	155 (27)
**CCB**	79 (30)	54 (24)	23 (26)	0.318	156 (27)
**Nitrates**	20 (8)	27 (12)	28 (32)	0.001*	75 (13)
**Insulin**	29 (11)	39 (18)	18 (21)	0.036*	86 (15)
**Metformin**	192 (74)	140 (63)	56 (64)	0.027*	388 (68)
**Gliclazide**	49 (19)	46 (21)	16 (18)	0.821	111 (20)
**SGLT2**	28 (11)	6 (3)	6 (7)	0.003*	53 (9)
**GLP-1**	28 (11)	35 (16)	13 (15)	0.250	76 (13)
**Gliptin**	23 (9)	6 (3)	1 (1)	0.002*	30 (5)

*P* value is considered significant at <0.05 and indicated by*. Variables are presented as numbers (%).

MPR, myocardial perfusion reserve; LGE, late gadolinium enhancement; MI, myocardial infarction; DOAC, direct oral anticoagulant; ACEI, angiotensin converting enzyme inhibitor; ARB, angiotensin receptor blocker; ARNI, angiotensin receptor-neprolysin inhibitor; MRA, mineralocorticoid receptor antagonist; CCB, calcium channel blocker; SGLT2, sodium glucose co-transporter 2 inhibitor; GLP-1, glucagon-like peptide-1 agonist.

Interestingly these patients were more frequently prescribed metformin, SGLT-2 inhibitors and gliptins compared to the other two groups.

#### MI+ or abnormal MPR T2DM patients

Patients with T2DM who had evidence of MI or abnormal MPR had a higher prevalence of hypertension than those with no MI and normal MPR but lower than those in the MI+ or abnormal MPR group. The rate of prescribed antiplatelet agents and heart failure medications (including beta blockers, ACEI, ARB, ARNI, MRA or diuretics) was higher than in those with no MI and normal MPR and lower than in those in the MI+ and abnormal MPR group (*Table 2*).

#### MI+ and abnormal MPR T2DM patients

Patients with MI+ and abnormal MPR were older and had a higher prevalence of previous MI, CABG surgery or PCI compared to the other two groups (*Table 1*). They also had a higher frequency of previous stroke or TIA, hypertension and peripheral vascular disease compared to the other two groups (*Table 1*). These patients were more frequently prescribed heart failure medications (including beta blockers, ACEI, ARB, ARNI, MRA or diuretics) compared to the other groups (*Table 2*). They were also more likely to be prescribed antiplatelet medication, statin treatment and angina medicine such as nitrates than those participants in the other two groups.

#### Healthy controls

Subjects in the healthy control group (n = 52) were not taking any medications. They had a mean age of 64 ± 6 years, and 65% of them were of male sex (*Table 1*).

### CMR assessment

Of the 572 patients scanned, 403 patients (70%) were scanned on 3T, and 169 patients (30%) were scanned on 1.5T CMR scanners. There were statistically significant differences in stress MBF, rest MBF and MPR between the two field strengths (Supplementary data online, *Table S2A*). No significant differences in stress MBF, rest MBF, or MPR were found between FLASH vs. bSSFP at 3T (Supplementary data online, *Table S2B*).

The mean stress MBF of the healthy control group was 2.05 ± 0.4 mL/g/min, and the mean MPR was 3.40 ± 0.7. Stress MBF and MPR in controls were significantly higher compared to patients (*P* < 0.0001 and *P* < 0.0001, respectively). None of the healthy controls had evidence of silent MI or non-ischaemic scar on LGE CMR. The threshold for abnormal MPR derived from the control group was MPR <1.91 (i.e. 3.40–2 × Standard Deviation).

#### Patients with T2DM

The mean LVEF across all patients with T2DM was 57 ± 15% (*Table 3*). On LGE, 29% patients had evidence of previous MI. The mean global stress MBF in all participants was 1.70 ± 0.6 mL/g/min, and the mean MPR was 2.20 ± 0.90.

**Table 3:** CMR parameters divided into groups

CMR Parameter	MI− and normal MPR (n = 261)	MI+ or abnormal MPR (n = 223)	MI+ and abnormal MPR (n = 88)	*P* value	All patients (n = 572)
**LVEF (%)**	62 ± 13	54 ± 16	51 ± 17	<0.001*	57 ± 15
**LVEDV (mL)**	144 ± 50	155 ± 44	173 ± 60	<0.001*	157 ± 59
**LVEDV index (mL)**	69 ± 17	74 ± 24	85 ± 31	<0.001*	73 ± 23
**LV mass (g)**	113 ± 32	122 ± 36	137 ± 40	<0.001*	120 ± 36
**LV mass index (g)**	52 ± 17	55 ± 18	61 ± 27	*0.02	55 ± 20
**Global Stress MBF (mL/g/min)**	1.88 ± 0.6	1.64 ± 0.5	1.31 ± 0.4	<0.001*	1.7 ± 0.6
**Rest MBF (mL/g/min)**	0.66 ± 0.27	0.87 ± 0.67	0.90 ± 0.48	<0.001*	0.8 ± 0.4
**MPR**	2.81 ± 0.7	1.78 ± 0.7	1.41 ± 0.4	<0.001*	2.2 ± 0.9
**Ischaemic LGE**	0	76 (34)	88 (100)	<0.001*	164 (29)
**Non-ischaemic LGE**	90 (35)	49 (22)	0	<0.001*	139 (24)
**No LGE**	171 (66)	98 (44)	0	<0.001*	267 (47)

*P* value is considered significant at <0.05 and indicated by *. ANOVA is used for parametric data, and Kruskal-Wallis for non-parametric data. Continuous variables are presented as mean ± SD or median + IQR, depending on normality. Dichotomous variables are presented as a number (%).

LGE, late gadolinium enhancement; MI, myocardial infarction; MBF, myocardial blood flow; MPR, myocardial perfusion reserve; LV, left ventricle; LVEF, left ventricular ejection fraction; LVEDV, left ventricular end-diastolic volume.

Patients in the MI− and normal MPR group had the highest LVEF, lowest EDV, lowest myocardial mass, and the highest level of stress MBF and MPR. On LGE, 35% of these patients had a non-ischaemic pattern. Those in the MI+ or abnormal MPR group had modest LVEF, LVEDV, myocardial mass and myocardial perfusion, whereas participants in the MI+ and abnormal MPR group had the lowest LVEF, highest LVEDV, highest myocardial mass and, on quantitative perfusion analysis, the lowest stress MBF and MPR (*Table 3*).

### Outcomes

**Figure 2:**
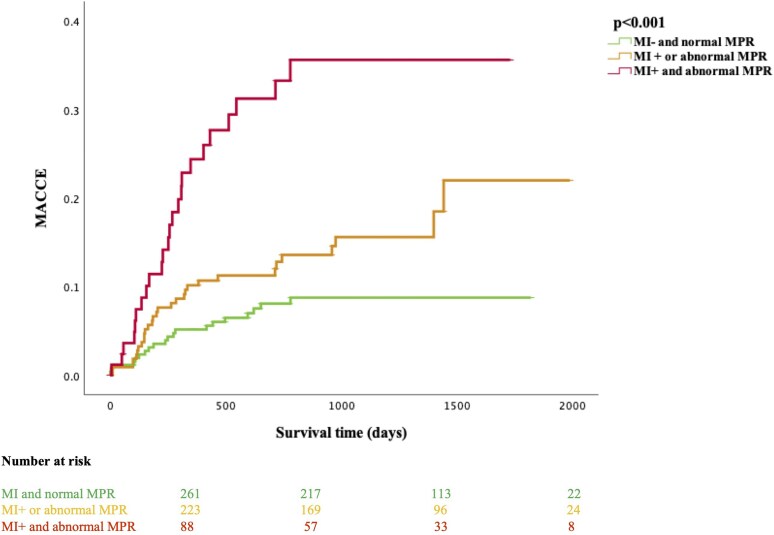
Kaplan-Meier hazard curves and number at risk for major adverse cardiovascular and cerebrovascular events (MACCE) divided into 3 groups. The red line represents MI+ and abnormal MPR, the orange line represents MI+ or abnormal MPR, and the green line represents MI− and normal MPR. MI, myocardial infarction; LGE, late gadolinium enhancement; MPR, myocardial perfusion reserve.

Overall, 81 participants accrued at least one MACCE (14%). Total MACCE included 25 deaths, 15 MIs, 18 strokes, 20 heart failure hospitalizations and 31 late coronary revascularizations (either PCI or CABG surgery >90 days after the CMR scan). Kaplan-Meier analysis showed higher event rates for MACCE in the MI+ and abnormal MPR group, followed by the MI+ or abnormal MPR group and the lowest event rate in those with no MI and normal MPR (*Figure 2*). Patients in the MI+ and abnormal MPR group had a MACCE rate of 30%. All cause death, MI and heart failure hospitalizations were statistically different between groups, but stroke events and coronary revascularization events were not (*Table 4*). Those in the MI+ or abnormal MPR group had a MACCE rate of 15%, and patients who had no MI and normal MPR had the lowest event rate of 8%. Supplementary data online, *Figure S1* shows the Kaplan-Meier analysis for MACCE, excluding cerebrovascular events, illustrating a similar distribution of events between the groups as the main analysis.

**Table 4:** MACCE events divided into groups

	MI− and normal MPR (n = 261)	MI+ or abnormal MPR (n = 223)	MI+ and abnormal MPR (n = 88)	*P* value	All patients (n = 572)
**MACCE**	22 (8)	33 (15)	26 (30)	<0.001*	81 (14)
**All-cause mortality**	5 (2)	12 (5)	8 (9)	0.011*	25 (4)
**MI**	3 (1)	5 (2)	7 (8)	0.002*	15 (3)
**Stroke**	8 (3)	9 (4)	1 (1)	0.417	18 (3)
**Heart Failure hospitalization**	4 (2)	6 (3)	10 (11)	<0.001*	20 (4)
**Coronary revascularization**	11 (4)	13 (6)	7 (8)	0.384	31 (5)

*P* value is considered significant at <0.05 and indicated by *.

MACCE, major adverse cardiovascular and cerebrovascular events; MI, myocardial infarction; MPR, myocardial perfusion reserve.

Supplementary data online, *Tables S3*  *and S4* show CMR parameters and MACCE separately for MI ± and normal/abnormal MPR groups.

Patients who suffered MACCE were significantly older, more likely to be male, had a significantly lower LVEF, a higher proportion of ischaemic LGE, significantly lower stress MBF and MPR (Supplementary data online, *Table S5*). Patients who suffered a HF hospitalization were significantly older and had a significantly lower LVEF (mean 38 ± 19%), significantly higher LVEDV, significantly lower stress MBF and significantly lower MPR, compared with the entire cohort (Supplementary data online, *Table S6*). The majority of these patients also had ischaemic LGE (55%).

MI and abnormal MPR added incremental prognostic value on multivariate Cox regression analysis (*Table 5*). The adjusted HR for MI alone was 2.42 (1.53–3.8), whereas for the combination of MI+ and abnormal MPR, the adjusted HR was much greater at 3.24 [95% CI 1.75–6.01], *P* < 0.001. For MI+ or abnormal MPR, adjusted HR was 1.86 [95% CI 1.06–3.25], *P* = 0.03. Non-ischaemic LGE (not shown in *Table 5*) was not associated with prognostic significance on univariate, HR 0.70 (95% CI 0.35–1.35), *P* = 0.28 or multivariable Cox regression after adjustment for age, sex, LVEF, LVEDV, LV mass: HR 0.58 (95% CI 0.29–1.14), *P* = 0.12. Net reclassification improvement and integrated discrimination improvement results both showed that adding MPR to LGE provides significant incremental prognostic value (*Table 6*).

**Table 5:** Univariate and multivariate Cox proportional hazard models

	Univariate			Multivariate^a^		
Unadjusted HR	Hazard Ratio	CI	*P* value	Hazard Ratio	CI	*P* value
**Stress MBF**	0.49	0.32–0.75	<0.001*	0.54	0.34.0.86	0.009*
**MPR**	0.70	0.54–0.91	0.008*	0.73	0.56–0.98	0.036*
**Rest MBF**	0.86	0.57–1.30	0.48	0.89	0.58–1.35	0.57
**MI on LGE**	2.80	1.8–4.3	<0.001*	2.42	1.53–3.8	<0.001*
**Globally reduced MPR**	1.95	1.24–3.05	0.004*	1.62	1.00–2.63	0.049*
**MI+ or abnormal MPR**	1.83	1.07–6.89	0.028*	1.86	1.06–3.25	0.030*
**MI+ and abnormal MPR**	3.89	2.19–6.89	<0.001*	3.24	1.75–6.01	<0.001*

*P* value considered significant at <0.05 and indicated by *.

CI, 95% confidence intervals; MI, myocardial infarction; MBF, myocardial blood flow; MPR, myocardial perfusion reserve; MACCE, major adverse cardiovascular and cerebrovascular events; LVEF, left ventricular ejection fraction; LV mass, left ventricular mass; LVEDV, left ventricular end-diastolic volume.

^a^Multivariate data are adjusted for age, sex, LVEF, LV mass, and LVEDV.

**Table 6:** Net reclassification improvement (NRI) and integrated discrimination improvement (IDI) for the addition of MPR to LGE in MACCE risk prediction

	Value	CI	*P* value
**NRI**			
All	0.27	0.04–0.51	0.024*
Event	0.16	−0.05 to 0.38	0.144
Non-event	0.11	0.02–0.20	0.017*
**IDI**			
All	0.02	0.01–0.03	0.002*
Event	0.01	0.004–0.025	0.006*
Non-event	0.003	−0.0008 to 0.0062	0.132

*P* value considered significant at <0.05 and indicated by *.

CI, confidence interval; MACCE, major adverse cardiovascular and cerebrovascular events; MPR, myocardial perfusion reserve; LGE, late gadolinium enhancement.

## Discussion

This multicentre study shows that in patients with T2DM, abnormal MPR and MI, as defined by CMR, are associated with increased MACCE. Patients with both pathologies have significantly higher cardiovascular event rates than those who have only one or neither of these two pathologies. Non-invasive assessment of quantitative myocardial perfusion and MI by CMR offers potential for future risk stratification in patients with T2DM.

### Prognostic value of myocardial blood flow and myocardial perfusion reserve

Global myocardial perfusion is a composite measure of the effects of epicardial CAD and coronary microvascular dysfunction (CMD). CMD is defined by impaired flow augmentation in response to pharmacological vasodilatation in the presence of non-obstructive CAD^22^ and heralds an increased risk of MACE.^23^ Global quantitative myocardial perfusion by CMR has been shown to provide independent prognostic value in other cohorts. In a multicentre study of 1049 patients with known or suspected CAD, reduced global stress MBF and MPR, measured using the same artificial intelligence-generated CMR method as in our current study, were strong, independent predictors of adverse cardiovascular outcome.^12^ This previous study included 298 patients with DM, who suffered 73/174 (42%) of all MACE, and the incidence of DM was significantly associated with the risk of MACE (*P* < 0.0001). Similarly, coronary flow reserve (CFR) < 2 derived from quantitative myocardial perfusion PET has been associated with adverse cardiovascular outcomes.^9,24^ In a study by Herzog *et al*., 18/229 had DM, and a diagnosis of DM was associated with a significantly increased risk of MACE, HR 1.94 (95% CI 1.14–2.87), *P* < 0.05. Another retrospective PET study of 2783 patients (1172 diabetics and 1611 non-diabetics) showed that patients with DM with preserved CFR had cardiac event rates similar to those of non-diabetics.^8^ A recent PET study looked at over 8000 patients referred for PET and found that higher rest MBF was associated with an increased risk of death and HF hospitalization.^25^In addition, a CMR study found that patients with dilated cardiomyopathy (DCM) exhibit CMD and, using quantitative perfusion CMR, showed they had significantly higher resting MBF compared to healthy controls.^26^ It is plausible that the higher resting MBF found in DCM patients and our patients with MI and reduced MPR may be secondary to increased haemodynamic load and structural alteration of the coronary microvasculature.^27^ Our data are in accordance with these previous studies, confirming that globally reduced MPR (both through reduced stress MBF and raised resting MBF) on quantitative myocardial perfusion CMR identifies cardiovascular risk in a contemporaneous cohort of patients with T2DM. Since quantitative myocardial perfusion CMR can today be readily integrated into standard scan protocols, is becoming more widely available and does not involve ionizing radiation, it may offer a more accessible route to wider adoption in routine care than quantitative PET perfusion.

### Prognostic value of ischaemic scar

Patients with T2DM and ischaemic scar (MI) are at a higher risk of cardiovascular mortality and adverse outcomes.^28^  ^,29^ In a previous CMR study of 187 diabetic patients,^7^ the presence of silent MI was associated with a threefold hazard increase for MACE and death (HR 3.71 and 3.61, *P* < 0.001 and *P* = 0.007, respectively). Furthermore, diabetic patients with silent MI had a cardiac event rate similar to their diabetic counterparts with clinical evidence of previous MI. This study found that LGE was the strongest and only significant multivariable predictor of MACE and mortality. In another CMR study, the presence of MI detected with LGE was found to be a predictor of adverse cardiovascular events, and this risk extended to patients with pre-diabetes.^30^ Our study is consistent with these previous results and confirms that patients with ischaemic scar (MI) had higher event rates than patients with abnormal quantitative myocardial perfusion alone or with non-ischaemic scar (Supplementary data online, *Table S7*). The detection of silent MI is of high clinical relevance in view of the associated high risk of adverse cardiovascular outcomes.^31,32^

In addition to detecting MI, LGE CMR also depicts focal non-ischaemic fibrosis, which is frequently seen in patients with DM. The prevalence of non-ischaemic scar in the literature ranges from 7–32%,^33–36^ in line with the prevalence in our study (30%). The prognostic relevance of non-ischaemic fibrosis has not been widely studied and a recent meta-analysis of the association of LGE with clinical outcomes in patients with DM was unable to assess the relationship between non-ischaemic LGE and MACCE/MACE due to lack of available data.^37^ However, a substudy of the ICELAND MI cohort reported a significant association of non-ischaemic LGE with the risk of heart failure and death.^38^ Our data showed no significant associations between non-ischaemic LGE and outcomes, but a trend towards prognostic relevance, which suggest further studies are warranted.

### Prognostic value of abnormal MPR and MI

Globally reduced MPR and MI have previously been shown to have independent prognostic implications in patients with T2DM. Our data shows that the risk of adverse events is amplified in patients who have both reduced MPR and MI compared with either of these findings alone. CMR is the only imaging modality able to quantify MBF and directly assess myocardial scar at high resolution, increasing sensitivity over other imaging modalities.^13–15^ In the stress CMR perfusion imaging in the United States study (SPINS) the prognostic relevance of a regional perfusion defect, suggesting epicardial CAD, and LGE was additive.^39^ Our study extends these findings to globally reduced MPR as a marker of CMD in a dedicated cohort of patients with T2DM. These findings indicate that risk from previous ischaemic injury and ongoing impairment of myocardial blood supply, either from epicardial or microvascular disease, are independent and cumulative markers of future cardiovascular risk.

### The role of medication

Interestingly, patients with T2DM who had no MI and normal MPR were more frequently prescribed metformin, SGLT-2 inhibitors and gliptins than those in the MI+ and/or abnormal MPR groups (*Table 2*). This study has not focused on the association between metformin, SGLT-2 inhibitors, gliptins and cardiovascular outcomes, although it is noteworthy, particularly since there is extensive work in the literature proving the beneficial effects of SGLT-2 inhibitors,^40–42^ metformin^43–45^ and gliptins^46^ in preventing cardiovascular events for patients with T2DM.

### Clinical relevance

The results observed in this study are clinically relevant for several reasons. First, we have found that globally reduced MPR as a surrogate marker for myocardial ischemia and/or CMD by CMR is independently predictive of cardiovascular outcomes in patients with T2DM. This suggests quantitative myocardial perfusion stress testing should be considered in patients with T2DM for optimal risk stratification, and those with globally reduced MPR should be offered intensive risk factor modification and rigorous medical therapy. Quantitative myocardial perfusion CMR is rapidly emerging as a diagnostic tool, and the quantification method used here is already available in over 100 centres worldwide. It uses an inline artificial intelligence-based approach which facilitates its integration into routine clinical care, even in less experienced centres. We have previously reported on the variability of the quantitative perfusion method and found no significant difference between stress or rest MBF measurements between centres when using age and sex matched samples.^47^

Compared with PET, CMR is a much lower cost test and avoids the use of ionizing radiation, making it more suitable for wider screening and risk stratification programmes.^48^ Furthermore, CMR is the most sensitive test to identify silent MI and the combined assessment of perfusion and MI makes it a powerful risk predictor, as shown in this study. In addition, in this study, we used global values of perfusion (including infarcted segments) to define globally reduced MPR, as used by Knott *et al*.,^12^ which is a simple and rapid measurement that can be easily integrated into routine clinical practice. While the calculation of perfusion values after exclusion of infarcted myocardium may be considered more physiologically consistent, it is time-consuming and hampered by the differences in spatial resolution and coverage of perfusion and LGE images and thus less likely to be used routinely. We also used MPR rather than stress MBF to define abnormal MPR. MPR values, as a ratio of stress and rest MBF, are much more consistent between different acquisition and post processing methods than stress MBF, making our results widely applicable.^49^ Although there is no generally accepted definition of abnormal MPR our cut off value of MPR <1.91 correlates well with the current evidence and literature.^12,50^

#### Limitations

Since this is an observational study, the associations found do not imply causation and there is potential for bias in the estimated coefficients. Due to the relatively small number of events, and the importance of not ‘over fitting’ the Cox model, we could only adjust our model to a limited number of variables, and chose the most widely reported predictors of outcome, age, sex, LVEF, LV mass and LVEDV. Other potential confounders that were positive in univariate analysis, including other demographics, could not be adjusted for in the multivariate model. One quarter of patients had evidence of previous coronary artery disease. However, since we did not undertake CTCA or invasive coronary angiography on the remaining 75%, we cannot reliably conclude these patients did not have coronary artery disease. Furthermore, we included patients scanned on both 1.5T and 3T and found significant differences between stress MBF and MPR between field strengths. It cannot be determined if these differences relate to differences in the study populations or field strength and future studies specifically designed to compare QP CMR at different field strengths are needed to explore this question in more detail. Further assessment for heart failure such as 6-minute walk tests or natriuretic peptide testing was not undertaken in this study. Furthermore, since events were documented using electronic patients records from different hospitals, there is a chance that events may have been missed to follow up. We only included patients with more than one year follow up, which may have led to bias related to patients who died or lost to follow up before their 12-month review. Patients with contraindications to gadolinium (for example patients with end stage renal disease) and those with contraindications to adenosine e.g. severe asthma, were excluded from this study. This study was undertaken partway through the COVID-19 pandemic. The impact of COVID-19 was not specifically investigated in this study but may have affected our data in several ways. COVID-19 generally meant that fewer patients presented to hospital which may have limited the number of MACCE in this study. At the same time, COVID-19 itself may have led to a number of events. The use of listwise deletion for missing data should also be acknowledged as a limitation. A further limitation is that the analyses presented in this study are retrospective which may have given rise to recruitment bias.

## Conclusion

In individuals with T2DM, the presence of globally reduced MPR on quantitative myocardial perfusion or MI on LGE CMR are associated with MACCE and the presence of both offers strong incremental prognostic value.

## Supplementary Material

jeag047_Supplementary_Data

## Data Availability

The datasets used and analysed during the current study are available from the corresponding author on reasonable request.
