# Supplementary material for: Abnormal myocardial perfusion reserve and myocardial infarction determine cardiovascular outcomes in type 2 diabetes mellitus
Source: Eur Heart J Cardiovasc Imaging. 2026 Feb 25;27(5):984–95. doi: 10.1093/ehjci/jeag047 (PMC13128276; doi:10.1093/ehjci/jeag047)
Supplement: jeag047_Supplementary_Data [file jeag047_supplementary_data.docx]

**Supplement- EHJ_CVI**

Supplementary table S1: Indications for stress perfusion CMR

| **Indication for CMR** | **Number (%)** |
| --- | --- |
| Chest pain | 154 (27) |
| Shortness of breath | 67 (12) |
| Aetiology of heart failure | 112 (20) |
| Viability assessment | 15 (3) |
| Research | 224 (39) |

Dichotomous variables are presented as number of patients (%).

Supplementary Table S2A: Stress MBF, rest MBF and MPR according to scanner field strength

| **Parameter** | **1.5T (n=169)** | **3T (n=403)** | **P value** |
| --- | --- | --- | --- |
| **Stress MBF (ml/g/min)** | 1.79± 0.6 | 1.66±0.5 | 0.02* |
| **Rest MBF** | 0.88±0.47 | 0.70±0.36 | <0.001* |
| **MPR** | 1.90±0.8 | 2.30±0.9 | <0.001* |

Continuous variables are presented as mean ± SD or median +IQR depending on normality. P value significant < 0.05 and indicated by *

MBF, myocardial blood flow; MPR, myocardial perfusion reserve; T, tesla.

Supplementary Table S2B: Stress MBF, rest MBF and MPR according to pulse sequence at 3T

| **Parameter** | **3T (n=403)** | | **P value** |
| --- | --- | --- | --- |
|  | **FLASH (n=309)** | **bSSFP (n=94)** |  |
| **Stress MBF (ml/g/min)** | 1.63±0.5 | 1.76±0.6 | 0.09 |
| **Rest MBF** | 0.68±0.4 | 0.80±0.3 | 0.44 |
| **MPR** | 2.32±1.0 | 2.24±0.7 | 0.50 |

Continuous variables are presented as mean ± SD or median +IQR depending on normality. P value significant at <0.05 and indicated by *

MBF, myocardial blood flow; MPR, myocardial perfusion reserve; FLASH, fast low angle shot imaging; bSSFP, balanced steady-state free precession imaging; T, tesla.

Table S3: CMR parameters according to presence or absence of MI and normal or abnormal MPR

| **CMR Parameter** | **MI- (n=408)** | **MI+ (n=164)** | **Normal MPR (n=326)** | **Abnormal MPR (n=246)** | **All patients (n=572)** |
| --- | --- | --- | --- | --- | --- |
| **LVEF (%)** | 59±15 | 53±15 | 60±13 | 53±17 | 57±15 |
| **LVEDV (ml)** | 151±56 | 171±63 | 151±55 | 168±62 | 157±59 |
| **LVEDV index (ml)** | 68±17 | 84±30 | 72±22 | 75±25 | 73±23 |
| **LV mass (g)** | 117±35 | 128±38 | 114±32 | 129±39 | 120±36 |
| **LV mass index (g)** | 53±17 | 57±23 | 53±18 | 58±22 | 55±20 |
| **Global Stress MBF (ml/g/min)** | 1.79±0.6 | 1.47± 0.5 | 1.83±0.6 | 1.51±0.5 | 1.7±0.6 |
| **Rest MBF** | 0.78±0.4 | 0.73±0.4 | 0.68±0.2 | 1.23±0.8 | 0.8±0.4 |
| **MPR** | 2.27±0.9 | 1.97±0.8 | 2.76±0.7 | 1.38±0.3 | 2.2±0.9 |
| **Ischemic LGE** | 0 | 164 (100) | 74 (23) | 90 (37) | 164 (29) |
| **Non-ischemic LGE** | 139 (34) | 0 | 88 (27) | 51 (21) | 139 (24) |
| **No LGE** | 269 (66) | 0 | 164 (50) | 105 (43) | 269 (47) |

Continuous variables are presented as mean ± SD or median +IQR depending on normality. Dichotomous variables are presented as number (%).

LV, left ventricular ejection fraction; LVEDV, left ventricular end diastolic volume; MBF, myocardial blood flow; MPR, myocardial perfusion reserve; LGE, late gadolinium enhancement.

Table S4: MACCE according to presence or absence of MI and normal or abnormal MPR

|  | **MI- (n=408)** | **MI+ (n=164)** | **Normal MPR (n=326)** | **Abnormal MPR (n=246)** | **All patients (n=572)** |
| --- | --- | --- | --- | --- | --- |
| **MACCE** | 42 (10) | 39 (24) | 33 (10) | 48 (20) | 81 (14) |
| **All-cause mortality** | 14 (3) | 11 (7) | 8 (2) | 17 (7) | 25 (4) |
| **MI** | 5 (10) | 10 (6) | 6 (2) | 9 (4) | 15 (3) |
| **Stroke** | 14 (3) | 4 (2) | 11 (3) | 7 (3) | 18 (3) |
| **Heart Failure hospitalisation** | 9 (2) | 11 (7) | 5 (2) | 15 (6) | 20 (4) |
| **Coronary revascularisation** | 16 (4) | 15 (9) | 17 (5) | 14 (5) | 31 (5) |

Data is presented as number (%).

MBF, myocardial blood flow; MPR, myocardial perfusion reserve; MACEE, major adverse cardiovascular and cerebrovascular events.

Table S5: Demographic and CMR data for patients with and without MACCE

|  | **MACCE**  **(n=81)** | **No MACCE (n=491)** | **P value** |
| --- | --- | --- | --- |
| **Age (years)** | 68±10 | 64±10 | <0.001* |
| **Male Sex** | 64 (79) | 313 (64) | 0.007* |
| **LVEF (%)** | 54±20 | 58±15 | 0.034* |
| **LVEDV (ml)** | 165±69 | 156±57 | 0.100 |
| **Ischemic LGE** | 39 (48) | 125 (26) | <0.001* |
| **Non-ischemic LGE** | 12 (15) | 127 (26) | <0.001* |
| **No LGE** | 30 (37) | 239 (49) | <0.001* |
| **Stress MBF (ml/g/min)** | 1.50±0.6 | 1.73±0.6 | <0.001* |
| **Rest MBF (ml/g/min)** | 0.81±0.38 | 0.75±0.4 | 0.408 |
| **MPR** | 1.89±0.8 | 2.23±0.9 | <0.001* |

Continuous variables are presented as mean+/- SD or median + IQR depending on normality. Dichotomous variables are presented as number (%). P value considered significant < 0.05 and indicated by *.

LV, left ventricular ejection fraction; LVEDV, left ventricular end diastolic volume; MBF, myocardial blood flow; MPR, myocardial perfusion reserve; LGE, late gadolinium enhancement.

Table S6: Demographic and CMR data for patients with HF hospitalisations.

| **Factor** | **HF hospitalisation**  **(n=20)** | **No HF hospitalisation**  **(n=552)** | **P value** |
| --- | --- | --- | --- |
| **Age (years)** | 73±11 | 64±10 | <0.001* |
| **Male Sex** | 12 (60) | 365 (66) | 0.570 |
| **LVEF (%)** | 38±19 | 58±15 | <0.001* |
| **LVEDV (ml)** | 213±87 | 155±56 | <0.001* |
| **LV Mass (g)** | 144 ±50 | 119±35 | 0.003* |
| **Stress MBF (ml/g/min)** | 1.25±0.4 | 1.71±0.6 | <0.001* |
| **Rest MBF** | 0.79±0.8 | 0.76±0.4 | 0.785 |
| **MPR** | 1.62±0.62 | 2.21±0.9 | 0.004* |
| **Ischemic LGE** | 11 (55) | 153 (28) | 0.03* |
| **Non- ischemic LGE** | 3 (15) | 136 (25) | 0.03* |
| **No LGE** | 6 (30) | 263 (48) | 0.03* |

Continuous variables are presented as mean ± SD or median +IQR depending on normality. Dichotomous variables are presented as number (%). P value considered significant < 0.05 and indicated by *

LV, left ventricular ejection fraction; LVEDV, left ventricular end diastolic volume; MBF, myocardial blood flow; MPR, myocardial perfusion reserve; LGE, late gadolinium enhancement.

Table S7: MACCE data by LGE distribution

|  | **Ischemic LGE (n=164)** | **Non -ischemic LGE (n=139)** | **No LGE (n=269)** | **P value** | **All patients (n=572)** |
| --- | --- | --- | --- | --- | --- |
| **MACCE** | 39 (24) | 12 (9) | 30 (11) | <0.001* | 81 (14) |
| **All-cause mortality** | 11 (7) | 3 (2) | 11 (4) | 0.154 | 25 (4) |
| **MI** | 10 (6) | 2 (1) | 3 (1) | 0.004* | 15 (3) |
| **Stroke** | 4 (2) | 3 (2) | 11 (4) | 0.465 | 18 (3) |
| **Heart Failure hospitalisation** | 11 (7) | 3 (2) | 6 (2) | 0.031* | 20 (4) |
| **Coronary revascularisation** | 15 (9) | 5 (4) | 11 (4) | 0.045* | 31 (5) |

Data is presented as number (%). P value significant at < 0.05 and indicated by *.

MACCE, major adverse cardiovascular and cerebrovascular events; MI, myocardial infarction; LGE, late gadolinium enhancement.

Figure S1: Kaplan-Meier curve excluding cerebrovascular events


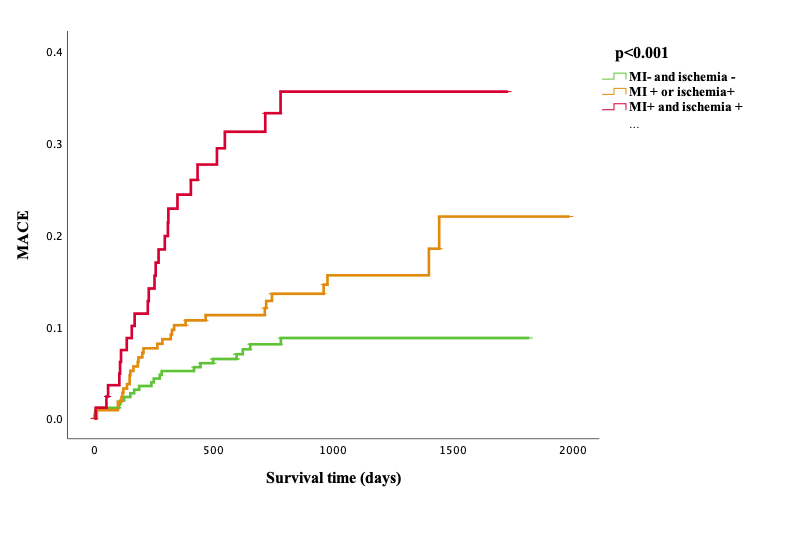


MACE, major adverse cardiovascular events.
